# Celecoxib Blocks Vasculogenic Mimicry *via* an Off-Target Effect to Radiosensitize Lung Cancer Cells: An Experimental Study

**DOI:** 10.3389/fonc.2021.697227

**Published:** 2021-09-10

**Authors:** Kai Niu, Xie-Wan Chen, Yu Qin, Lu-Ping Zhang, Rong-Xia Liao, Jian-Guo Sun

**Affiliations:** ^1^Cancer Institute of Chinese People's Liberation Army (PLA), Xinqiao Hospital, Army Medical University, Chongqing, China; ^2^Medical English Department, College of Basic Medicine, Army Medical University, Chongqing, China; ^3^Nutrition and Food Hygiene Department, Institute of Military Preventive Medicine, Army Medical University, Chongqing, China

**Keywords:** celecoxib, vasculogenic mimicry, radiosensitizing effect, off-target effect, lung cancer, cyclooxygenase-2, aminopeptidase N, integrin alpha-V

## Abstract

The resistance to radiotherapy in lung cancer can be attributed to vasculogenic mimicry (VM) to some extent. Celecoxib (CXB), a selective inhibitor of cyclooxygenase-2 (COX-2), is reported as a radiosensitizer in non-small cell lung cancer (NSCLC). However, whether CXB can regulate VM formation *via* an off-target effect to radiosensitize NSCLC remains unclear. This study aimed to elucidate the mechanism underlying the radiosensitizing effect of CXB on NSCLC, i.e., whether CXB can inhibit VM formation *via* binding to newly identified targets other than COX-2. CXB radiosensitivity assay was performed in BALB/c mice bearing H460 xenografts and C57 mice bearing Lewis lung cancer (LLC) xenografts, which were divided into the control, CXB, irradiation (IR) treatment, and IR plus CXB groups. VM formation was observed using 3D Matrigel, periodic acid solution (PAS) staining, and immunofluorescence staining. The potential off-targets of CXB were screened using Protein Data Bank (PDB) database, MGLTools 1.5.6, and AutoDock Vina 1.1.2 and confirmed by Western blotting, enzyme activity assay, and RNA interference *in vitro* experiments and by immunohistochemistry *in vivo* experiments. CXB treatment almost eliminated the enhancement of VM formation by IR *in vitro* and *in vivo*, partially due to COX-2 inhibition. Four potential off-targets were predicted by molecular docking. Among them, aminopeptidase N (APN) and integrin alpha-V (ITAV) were remarkably inhibited in protein expression and enzyme activity *in vitro* or *in vivo*, consistent with the remarkable reduction of VM formation in H460 xenografts in BALB/c mice. In conclusion, CXB dramatically blocked VM through inhibiting newly identified off-targets APN and ITAV, other than COX-2, then radiosensitizing NSCLC.

## Introduction

Celecoxib (CXB), a selective inhibitor of cyclooxygenase-2 (COX-2), has shown capability of radiosensitization in several cancer cell lines in experimental studies in the last decades (1–3). The above effects are not always associated with COX-2 enzyme inhibition. Dittmann et al. (4) found that the radiosensitization effect of CXB was independent of the inhibition of COX-2 but related to mediating nuclear epidermal growth factor receptor (EGFR) transport and DNA repair. Another study also reported that c-Myc could be considered an off-target of CXB in its beneficial effect on cancer treatment (5). Therefore, we speculate that there may exist other potential off-targets of CXB contributing to its radiosensitization effect.

Studies conducted by Brown claimed that vasculogenesis, a process of the formation of blood vessels from circulating cells, plays a crucial role in the resistance of solid tumors to radiotherapy (6, 7). Other studies supported this claim by presenting that angiogenesis involving endothelial cells (ECs) sprouting from nearby blood vessels contributed to radiotherapy resistance (8, 9). Recently, vasculogenic mimicry (VM) was revealed as a novel mechanism underlying vasculature development in tumors, with the ability to form fluid-conducting structures in an EC-free manner by tumor cells themselves (10, 11).

Lung cancer is the leading cause of cancer deaths among men and women (12). Non-small cell lung cancer (NSCLC) is the most common type of lung cancer and has a really high level of tumor metastasis (13). A large number of NSCLC patients receive radiotherapy during the entire course of cancer treatment. However, it is often of tumor relapse and distant metastasis due to radiation resistance (14, 15). VM has been observed in NSCLC tissues in some studies (16–18). VM constituted an important factor for tumor relapse and distant metastasis in glioblastoma, breast, and lung cancers (19–22). However, the role of VM in radiation resistance and relapse of NSCLC remains unclear.

Tumor neovascularization targeted drugs including Avastin, Endostar, sorafenib, sunitinib, anotinib, apatinib have been used as the antitumor clinical practice in many solid tumors, including NSCLC (23) and hepatocellular carcinoma (24). However, these drug resistance indicates that there are some compensation for angiogenesis, including VM (25). Thus, if a drug can effectively inhibit VM, it has the potential to remove anti-angiogenesis resistance and improve clinical outcomes.

Previous studies demonstrated that phosphatidylinositol 3-kinase (PI3K) and vascular endothelial growth factor (VEGF), and COX-2 participated in VM formation (26–28). It was reported that inhibition of COX-2 by CXB led to impairment of VM formation in U87 cells and xenografts through downregulation of prostaglandin E2 and protein kinase C (28). Additionally, CXB was reported to destroy VM channels in brain glioma and breast cancer cells (29, 30). However, whether CXB exerts radiosensitization effects on NSCLC cells and xenografts *via* inhibiting VM formation and regulating off-targets needs to be elucidated.

In the current study, we investigated the effect of CXB on VM formation after irradiation (IR) treatment in lung cancer *in vitro* and *in vivo*. Furthermore, we explored the new off-targets of CXB in VM using molecular docking to clarify the mechanism by which CXB radiosensitizes NSCLC.

## Materials and Methods

### *In Vitro* Experiment and Irradiation Treatment

Human-derived NSCLC cell lines (A549, H460, HCC78, etc.) and mouse-derived Lewis lung cancer (LLC) cells were purchased from the Cell Bank of Typical Culture Preservation Committee of Chinese Academy of Sciences (Shanghai, China). As a mouse brain capillary EC line, bEnd.3 cell was used as a positive control of VM formation in this study (31). Cells were cultured in RPMI-1640 culture medium (Gibco, USA) supplemented with 10% fetal bovine serum (HyClone, USA), 100 U/ml of penicillin, and 100 µg/ml of streptomycin at 37°C with 5% CO_2_.

The influence of CXB at different doses (0–80 μg/ml) for 24 h or at a certain dose (0, 15, and 30 μg/ml) for 0–10 days on the cellular viability of A549 cells was assessed by CCK-8 test (Beyotime Biotechnology, China) according to the manufacturer’s instructions. The result of CCK-8 was used to identify the suitable dose of CXB for the following study.

The cells were assigned into the control group, CXB group, IR group, and IR plus CXB group. The CXB group was incubated with CXB (15 μg/ml from Sigma-Aldrich Corporation, Saint Louis, USA) for 24 h; and the IR group was exposed to X-ray (X-RAD 320, USA) at a dose of 5 Gy. The IR plus CXB group was treated with both CXB and X-ray. The control group was treated with 0.1% dimethyl sulfoxide (DMSO). IR was delivered at a dose rate of 2,058 cGy/min with 35-cm height.

### *In Vivo* Experiment and Irradiation Treatment

BALB/c nude mice and C57 mice (aged 6–8 weeks) were supplied by Animal Laboratory Center of Daping Hospital (Chongqing, China). A total of 2 × 10^6^ cells at passage 3 after cell recovery was resuspended in 100 μl of physiological saline solution and subcutaneously injected into the right hind limb of BALB/c nude mice and C57 mice, to build xenograft models. After 7 to 10 days, the tumor tissue had grown to about 7 mm in diameter, and then BALB/c nude mice and C57 mice were randomly assigned to the control, CXB, IR, and IR plus CXB groups (*n* = 6 each). C57 mice in the CXB group received CXB suspended in maize oil at a dose of 50 mg/kg·bw by gavage for 5 days. The above exposure dose was calculated based on AUC0-24 and equivalent to the clinical dose of about 200 mg twice daily (32). BALB/c mice in the CXB group were given CXB in drinking water at a dose of 15 µg/ml for 4 weeks. The IR group received 8 Gy of IR per day for 3 days continually for C57 mice and BALB/c nude mice. The IR plus CXB group received IR and CXB treatment simultaneously. The control group received maize oil by gavage or drinking water without CXB for C57 and BALB/c mice. After being intraperitoneally injected with procaine for anesthesia and fixed with leg jig and put into the irradiator, mice were vertically irradiated with X-rays (X-RAD 320, USA) only in the field of the right hind limb. The IR was delivered at a dose rate of 4,518 cGy/min with 45-cm height. Tumor tissues were collected when the mice displayed morbidity or the tumor was with ulcer. Survival experiment was also assessed using C57 mice with the same grouping described as above (five mice in each group). On the 50th day, all mice were sacrificed by cervical dislocation. All animal procedures were performed with the approval of the Laboratory Animal Welfare and Ethics Committee (SYXK-PLA-20120031) of Army Medical University (Chongqing, China).

### Assay of Vasculogenic Mimicry Channels *In Vitro*

The three-dimensional culture of Matrigel (BD Biosciences Corporation, USA) was used to simulate the formation of VM channels of lung cancer *in vitro*. Briefly, the Matrigel low growth factor matrix was melted into liquid at 4°C. Matrigel of 300 μl was added per well in the pre-cooled 24-well cell culture plate that was placed on ice. Then, the culture plate was placed at 37°C for 30 min to curdle the Matrigel. After solidification, A549, H460, HCC78, and bEnd.3 cells were seeded on the Matrigel. After incubating for 24 h, cells were assigned into the control group, CXB group, IR group, and IR plus CXB group and received corresponding treatments, followed by incubation at 37°C for 7 h. The effects of different treatments on the VM channels of lung cancer cells were observed with an inverted microscope (OMI 3000B, Leica, Germany).

### Cyclooxygenase-2 Expression in Tumor Tissues and Cells

COX-2 expression in tumor tissues from BALB/c nude mice was assayed by immunofluorescence staining. Rat anti-mouse COX-2 monoclonal antibody (Mcox-2, Abcam), and Alexa Fluor^®^ 488 rabbit anti-rat secondary antibody and Alexa Fluor^®^ 593 goat anti-rat secondary antibody (both from Abcam) were used. Nuclei were stained by 4′6-diamidino-2-phenylindole (DAPI). The experiments were carried out in triplicate. Images were acquired using a microscope (BX53, Olympus, Japan).

A549 and H460 cells were seeded in 24-well plates (1.5 × 10^4^ cells/well) pre-placed with sterilized glass slides and were assigned into the four groups. After CXB and IR treatment, a monolayer of cells was fixed with 4% (vol/vol) paraformaldehyde at 37°C for 10 min, followed by 10-min incubation at room temperature with 0.2% (vol/vol) Triton X-100. After being washed with phosphate-buffered saline (PBS), non-specific binding was blocked with 10% fetal bovine serum (FBS) in PBS. Anti-COX-2 rabbit polyclonal antibodies (1:50; Cell Signaling Technology) diluted in PBS were added at 4°C and incubated overnight, avoiding light; and then after being washed with PBS, the slides were incubated with DyLight^®^ 594 conjugated secondary antibody (Thermo Fisher, USA) in PBS for 1 h at room temperature. Finally, the slides were mounted in 0.1% (wt/vol) DAPI (1:50; Invitrogen) for nuclear counterstaining for 5 min at room temperature and observed under a Leica OMI 3000B fluorescence microscope (Leica, Germany).

COX-2 protein expressions in cells were tested by Western blotting, using the primary and secondary antibodies from Abcam (USA).

### Celecoxib Relocation and Cyclooxygenase-2-Independent Target Screening

The first step was identification of receptor protein database. Based on Qiagen’s gene pool involved in neovascularization, we selected 84 genes from an angiogenesis PCR chip, including genes for growth factors and their receptors, chemokines, cytokines, cell substrates, adhesion molecules, proteases and their inhibitors, and transcription factors. The proteins expressed by these genes constituted a database of potential receptor proteins.

The second step was structure screening and structural pretreatment for potential receptor proteins. The 3D structure of each protein was retrieved in the Protein Data Bank (PDB) database and then screened. The screening criteria were as follows: first, proteins without experimental crystal structure data or with NMR structure data only were excluded; second, proteins that only reported the structure of the complex formed by the protein and small molecules were excluded. For each of the remaining candidate proteins, the most desirable structure was selected after comprehensively considering its crystal structure resolution, fragment length, functional regions, and active sites. The MGLTools 1.5.6 was used to treat the receptor protein structure, including adding polar hydrogen atoms, removing non-polar hydrogen atoms, and storing the file in.pdbqt format. The structural data of CXB were downloaded from the PubChem database and processed into.pdbqt format using MGLTools.

The third step was determination of molecular docking box. In the case of docking a small ligand molecule with a series of receptor proteins, a docking box (i.e., an energy grid of the atom probe) was placed centering on the entire protein structure. For example, the center of the AKT1 protein (PDB ID: 4EJN) was 32.054 × 41.233 × 13.569, and a rectangular box having a side length of 40 × 50 × 60 was set, so that the box contained the entire protein molecule. The docking distance of the docking box for all proteins was set to 1.0 A.

The final step was docking calculation. A semi-flexible docking method was used to treat protein receptors as rigid materials, while small molecule ligand conformations could vary. AutoDock Vina 1.1.2 was used as a molecular docking tool to determine the binding sites and affinities of CXB to protein receptors.

### Western Blotting

After being harvested, cells were lysed in radioimmunoprecipitation assay (RIPA) lysis and extraction buffer (Beyotime Biotechnology, China) at 4°C with protease inhibitors and phosphatase inhibitors (Beyotime Biotechnology, China). Protein concentrations were detected using a bicinchoninic acid protein assay kit (Beyotime Biotechnology, China). Samples containing the same amount of protein were separated using 8%–10% sodium dodecyl sulfate–polyacrylamide gel electrophoresis (SDS-PAGE) electrophoresis and then electrotransferred onto polyvinylidene fluoride membranes (Bio-Rad Laboratories, USA), followed by blocking with the blocking buffer (Beyotime Biotechnology, China). These membranes were incubated at 4°C overnight with the primary antibodies (Abcam, USA) and then incubated with the secondary antibodies (Abcam, USA). Signals of the protein bands were detected using an enhanced chemiluminescence system (Millipore, Billerica, MA, USA). Target proteins were visualized with the FluorChem HD2 system (ProteinSimple, USA).

### Aminopeptidase N Enzyme Activity Assay

Aminopeptidase N (APN) enzyme activity was measured by spectrophoto-metrically method using l-leucine-*p*-nitroanilide (Sigma-Aldrich Corporation, Saint Louis, USA) as an APN substrate. Whole-cell (A549 and H460) suspensions were prepared in test tubes and then washed with PBS. Thereafter, 5 × 10^5^ cells were resuspended in 200 μl of PBS in each well of a 96-well microplate, and the substrate was added at a final concentration of 1.6 mM. APN enzyme activity was estimated by measuring the absorbance at 405 nm using a microplate reader (LabSystems, Multiskan Bichromatic) every 15 min during incubation at 37°C.

### Lentivirus Transfection

Tumor cells were transfected with lentivirus to inhibit integrin alpha-V (ITAV) expression. On the first day, A549 and H460 cells were seeded into 12-well plates (4 × 10^4^ cells/well) and incubated for 24 h. On day 2, 1 μl of configured empty or ITAV vector virus transfection solution was added into the culture media per well and then incubated for 24 h. Transfection effect was observed under a fluorescence microscope on days 3–5. On day 6, well-transfected cells were selected and seeded into six-well plates to be incubated for 72 h. Cells that grew well were seeded into a 25-cm^2^ flask and subcultured. The lentivirus-transfected cells were seeded into a 3D-Matrigel-coated 96-well plate to observe VM formation. Images were acquired using a microscope (OMI 3000B, Leica, Germany).

### Vasculogenic Mimicry Formation Identification *In Vivo*

VM formation in cells was also assessed using periodic acid solution (PAS) method as previously described (33). In brief, tumor sections were incubated with 0.5% PAS for 10 min. After being rinsed with distilled water for 2–3 min, tumor sections were incubated with Schiff solution for 15–30 min in a dark chamber and rinsed again. Tumor sections were finally counterstained with hematoxylin. PAS staining showed that the vessel-like structure was composed of tumor cells and basal membrane (VM formation). Images were acquired using a microscope (BX53, Olympus, Japan).

### Vasculogenic Mimicry Formation Identification *In Vivo* by Immunofluorescence Staining

VM formation identification *in vivo* was also observed by immunofluorescence staining using rat anti-mouse CD31 monoclonal antibody (mCD31, Santa Cruz), Alexa Fluor^®^ 488 rabbit anti-rat secondary antibody and rabbit anti-human E-cadherin monoclonal antibody (hE-cad, Abcam), and Alexa Fluor^®^ 593 goat anti-rabbit secondary antibody (Abcam). The expression of mCD31 was taken as the average immunofluorescence density and analyzed using Image J software (Softonic International, Spain). The images were analyzed with Image-Pro Plus (IPP) software (Media Cybernetics, USA). VM channels were identified as hE-cad-positive (in red) and mCD3-negative1 (in green) channels. Images were acquired using a microscope (Olympus, BX53, Japan).

### Immunohistochemistry

The lung cancer tissues from C57 mice were prepared for paraffin tissue slices. The slices were baked at 60°C in an oven for 2 h followed by deparaffinization and then were repaired with antigen retrieval solution (Tris-EDTA buffer, pH 9.0). These slices were incubated with endogenous peroxidase blocking solution and then blocked with goat serum. Next, these slices were incubated with rabbit monoclonal antibody anti-ITAV (EPR 16800, dilution 1:500, Abcam, USA), rabbit monoclonal antibody anti-COX-2 (EPR12012, dilution 1:1,000, Abcam, USA), and rabbit monoclonal antibody anti-CD13 (EPR4058, dilution 1:1,200, Abcam, USA) overnight at 4°C. Thereafter, these slices were incubated with secondary antibody (MaiXin, China) followed by incubating with DAB color reagent (MaiXin, China). Finally, the slices were stained with hematoxylin somatic cell staining solution (MaiXin, China) and then air-dried and mounted. They were observed using a fluorescence microscope (Olympus, Japan). The images were analyzed with IPP software (Media Cybernetics, USA). Images were acquired using a microscope (Olympus, BX53, Japan).

### Statistical Analysis

The significance of difference among groups was determined by ANOVA test, and the difference between two groups was determined by SNK assay using IBM SPSS 21.0 (USA). The significance of difference in the average fluorescence intensity of COX-2 in cells between control and CXB groups was determined by *t*-test. The survival analysis was assessed by Kaplan–Meier method. *p*-Values <0.05 were considered statistically significant.

## Results

### Celecoxib Exerted Radiosensitizing Effect in Lung Cancer Xenografts

**Figure 1:**
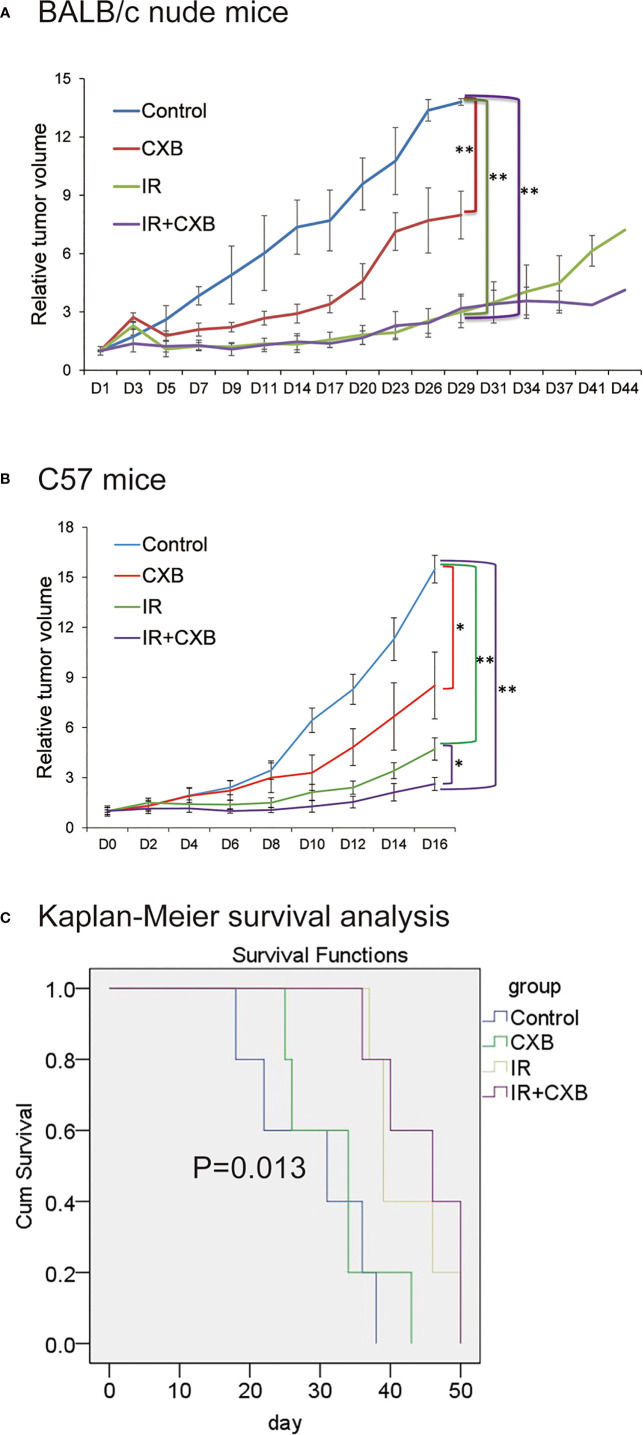
CXB had radiosensitizing effect. BALB/c nude mice and C57 mice were randomly assigned to the control, CXB, IR, and IR plus CXB groups. **(A)** The tumor growth curve of BALB/c nude mice injected with H460 cells (*n* = 6, each group). Compared with that in the control group, the tumor growth was significantly inhibited in IR plus CXB group in xenografts. Comparison with the control group: *p < 0.05 and **p < 0.01. **(B)** Tumor formation experiments in C57 mice bearing LLC xenografts (*n* = 6, each group). Data are expressed as the mean ± SD. The tumor growth was significantly inhibited in IR plus CXB group in xenografts. Comparison with the control group: ^*^p < 0.05 and ^**^p < 0.01. **(C)** Survival analysis of C57 mice bearing LLC xenografts, *p* = 0.013 among groups. CXB, celecoxib; IR, irradiation; LLC, Lewis lung cancer.

Compared with that in the control group, the tumor growth was significantly inhibited in IR plus CXB group in both BALB/c mice implanted with H460 cells and C57 mice implanted with LLC cells (*p* < 0.05; **Figures 1A, B**). In addition, CXB combined with IR remarkably prolonged the survival duration of mice with xenografts (*p* = 0.013; **Figure 1C**).

### Celecoxib Exerted Radiosensitizing Effect *via* Inhibiting Vasculogenic Mimicry Formation

**Figure 2:**
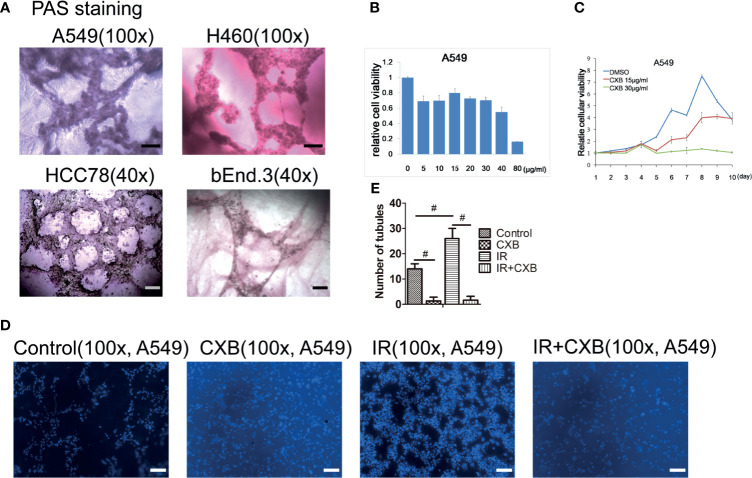
CXB exerted radiosensitizing effect *via* inhibiting VM formation. **(A)** VM formation in A549, H460, HCC78, and bEnd.3 cells grown on the 3D Matrigel in representative images with PAS staining. Scale bar, 500 μm. **(B)** The influence of CXB at different doses treated for 24 h on A549 cells analyzed by CCK-8 assay. CXB at a dose of 15 µg/ml did not dramatically inhibit the growth of A549 cells. **(C)** The influence of DMSO, and 15 and 30 µg/ml of CXB treatment for different durations on A549 cells analyzed by CCK-8 assay. CXB treatment at a dose of 15 µg/ml for 10 days did not dramatically inhibit the growth of A549 cells. **(D)** Pictures of tubules in different groups in A549 cells. **(E)** The number of tubules in different groups (the control, CXB, IR, and IR plus CXB groups) in A549 cells. Data are expressed as the mean ± SEM (*n* = 3). ^#^*p* < 0.001. Scale bar, 500 μm. CXB, celecoxib; VM, vasculogenic mimicry; PAS, periodic acid solution; DMSO, dimethyl sulfoxide; IR, irradiation.

*In vitro* tube-forming experiments showed that A549, H460, and HCC78 cells had an ability to form vessel-like structures with VM characteristics in 3D Matrigel (**Figure 2A**). To determine the concentration of CXB treatment, CCK-8 assays were performed. We found that 15 µg/ml of CXB incubation did not dramatically inhibit the growth of A549 cells for 24 h (**Figure 2B**) or for 10 days (**Figure 2C**). Thus, we chose 15 µg/ml as the treatment concentration of CXB for the following experiments. Meanwhile, IR treatment (5 Gy) significantly enhanced the ability of VM formation in all the three cell lines compared with the control group (data not shown). However, CXB treatment eliminated the formation of VM in A549 cells, whether with or without IR treatment (**Figures 2D, E**).

### Celecoxib Inhibited the Expression of Cyclooxygenase-2 in Cells and Xenografts

**Figure 3:**
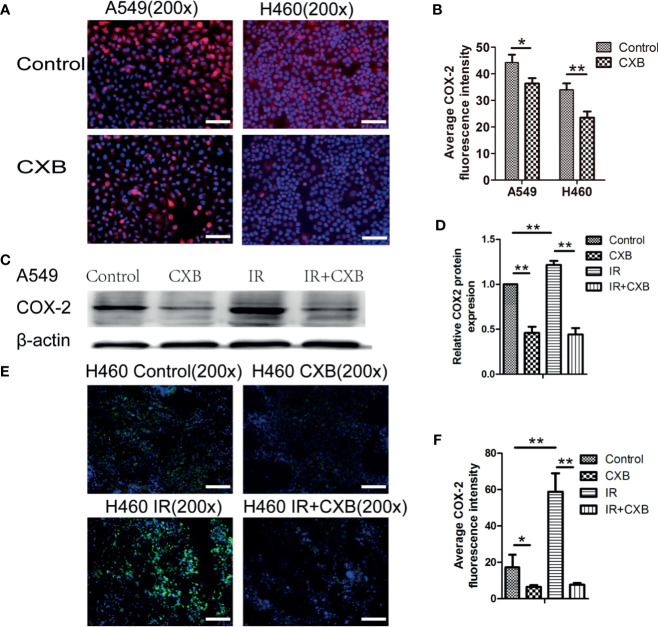
The expression of COX-2 in cells and the influence of CXB and/or IR on the expression of COX-2. **(A)** COX-2 immunofluorescence staining in A549 and H460 cells (blue, nucleus; red, COX-2). Scale bar, 100 μm. **(B)** Average fluorescence intensity of COX-2 in cells. Error bars indicate standard division. COX-2 expression levels in A549 and H460 cells were significantly decreased by CXB. *p < 0.05, **p < 0.01. **(C)** COX-2 protein expression in A549 cells analyzed by Western blotting (picture was cropped). **(D)** The relative COX-2 protein expression in A549 cells. **p < 0.01. **(E)** COX-2 immunofluorescence staining in tumor tissues from BALB/c nude mice (H460, *n* = 6; blue, nucleus; red, COX-2). Scale bar, 100 μm. **(F)** Average fluorescence intensity of COX-2 in tumor tissues from BALB/c nude mice (H460, *n* = 6). Error bars indicate standard division. *p < 0.05, **p < 0.01. COX-2, cyclooxygenase-2; CXB, celecoxib; IR, irradiation.

As shown in **Figures 3A, B**, COX-2 expression levels in A549 and H460 cells were significantly decreased by CXB. The COX-2 protein expression in A549 cells significantly decreased after CXB treatment, while IR treatment resulted in a remarkable increase in COX-2 expression (**Figures 3C, D**). Additionally, a combination treatment of IR and CXB suppressed the increment of COX-2 protein expression induced by IR (**Figures 3C, D**). In immunofluorescence staining assay, COX-2 expression was enhanced by IR treatment in H460 xenografts and noticeably reduced but not eliminated by CXB treatment with or without the combination of IR treatment (**Figures 3E, F**).

However, compared with the almost eliminatory effect on VM formation caused by CXB alone or combination with IR treatment, the reduction degrees in COX-2 protein expression and COX-2 immunofluorescence density in IR group were likely lower. These results suggest that CXB may perform an eliminatory effect on the formation of VM and enhance radiosensitivity not only *via* inhibiting COX-2 but also through interacting with other potential novel targets.

### Four Proteins (Aminopeptidase N, Integrin Alpha-V, AKT1, and NOS3) Were Screened as Potential Off-Targets of Celecoxib

**Figure 4:**
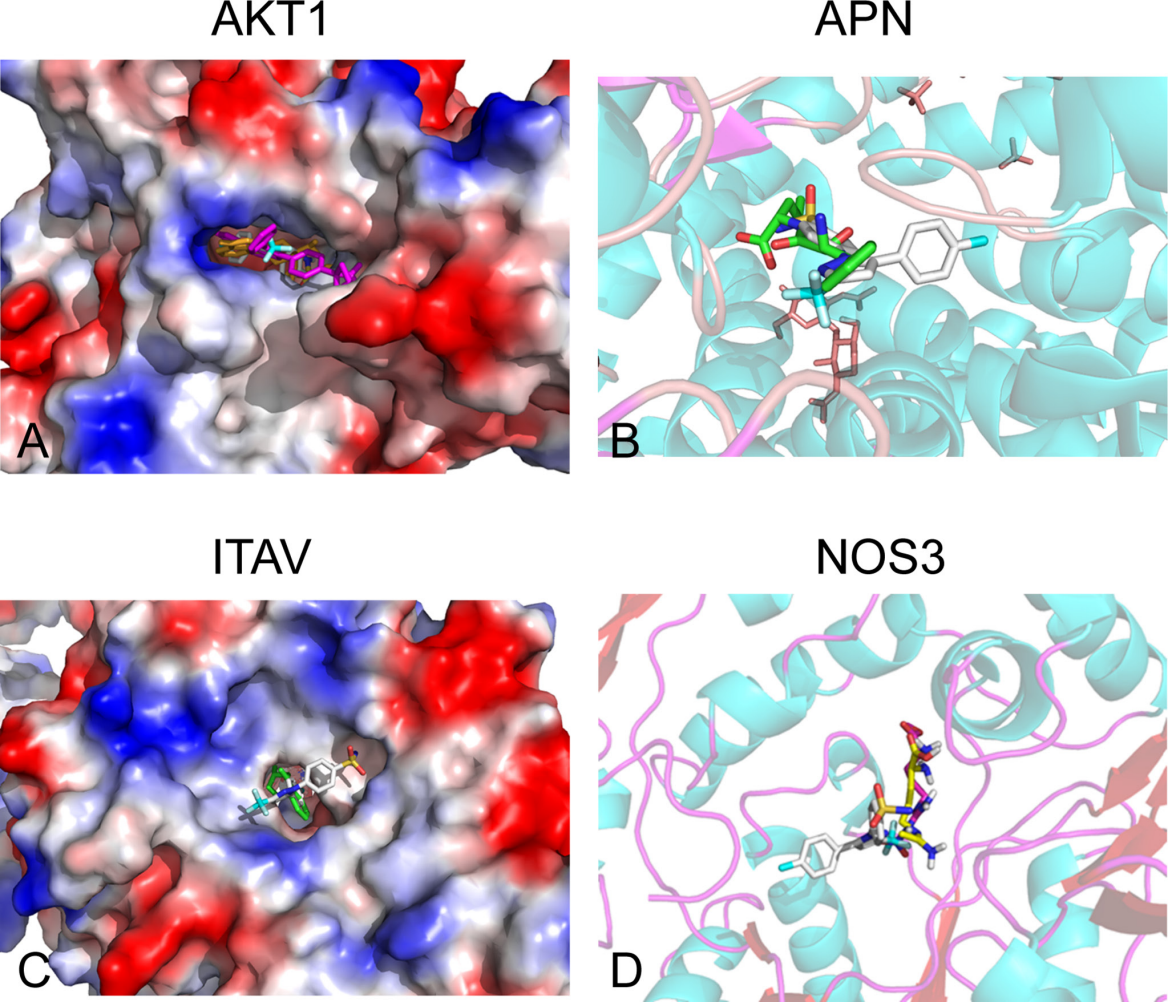
Off-targets screening for CXB by molecular docking. Four proteins (APN, ITAV, AKT1, and NOS3) were screened as potential off-targets of CXB. **(A)** AKT1 was aligned with CXB and MK-2206 and AZD5363 docking structures. Rose red, MK-2206; yellow, AZD5363; white, CXB. **(B)** APN was compared with CXB and bestatin (BST) docking structures. Translucent, APN; green, BST; white, CXB. **(C)** ITAV was aligned with CXB and endostatin and rapamycin docking structures. Green, endostatin; brown, rapamycin; white, CXB. **(D)** NOS3 was aligned with CXB and l-NAME and l-NIO docking structures. The translucent molecule is NOS3 (4D1O); rose, l-NAME; yellow, l-NIO; and white, CXB. CXB, celecoxib; APN, aminopeptidase N; ITAV, integrin alpha-V.

Of the 84 proteins involved in neovascularization, 54 had identified 3D structure. According to molecular docking, 21 proteins showed relatively high affinity with CXB, of which 14 had free binding energy of less than −8.0 kcal/mol and seven had less than −9.0 kcal/mol, particularly one with the lowest free binding energy of −10.6 kcal/mol (**Table 1**). Since free binding energy lower than −9.0 kcal/mol was considered of high affinity, we selected the seven proteins for further validation. To determine whether CXB could bind to the functional domain of the seven proteins, we compared the binding site and energy between CXB and reported inhibitor of each protein, MK-2206 or AZD5363 for AKT1, bestatin for APN, endostatin or rapamycin for ITAV, and l-NAME or l-NIO for NOS3. Comparative molecular docking revealed that CXB could bind to the functional domain of four proteins, APN, ITAV, AKT1, and NOS3 (**Figure 4**), among which APN showed the highest affinity.

**Table 1:** Molecular docking with free energy −9.0 kcal/mol and below as the high affinity standard screened out seven optimal binding proteins.

Receptor protein	PDB ID	Free binding energy (kcal/mol)
APN	4FYR	−10.6
AKT1	4EJN	−9.6
NOS3	4D1O	−9.6
SPHK1	4V24	−9.4
TGFA	3E50	−9.3
EPHB4	3ZEW	−9.3
ITAV	1JV2	−9.2

APN, aminopeptidase N; AKT1, RAC-alpha serine/threonine-protein kinase; NOS3, nitric oxide synthase, endothelial; SPHK1, sphingosine kinase 1; TGFA, protransforming growth factor alpha; EPHB4, ephrin type B receptor 4; ITAV, integrin alpha-V; PDB, Protein Data Bank.

### Aminopeptidase N and Integrin Alpha-V Were Identified as Off-Targets *In Vitro*

**Figure 5:**
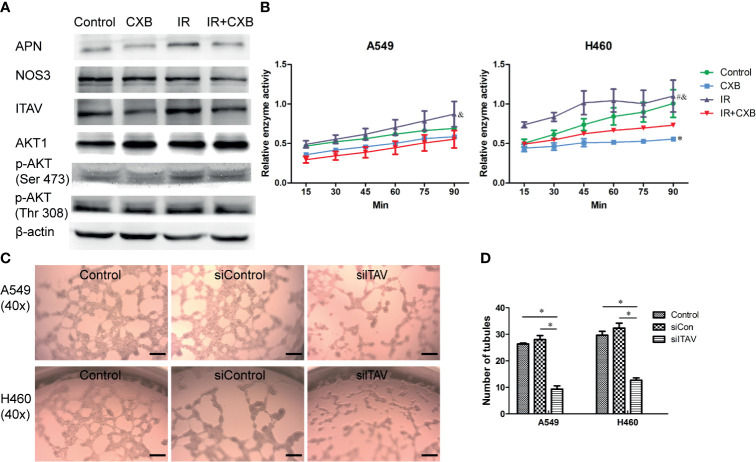
Confirmation of off-targets of CXB in cells. **(A)** Protein expression levels assessed by Western blotting (picture was cropped). Western blotting showed that IR significantly increased ITAV and APN levels, while CXB treatment significantly decreased ITAV and APN levels. Furthermore, IR plus CXB treatment remarkably reduced the increment of ITAV and APN levels induced by IR. **(B)** Enzyme activity test. APN enzyme activity experiment in cells (A549 and H460). *Comparison with the control group, *p* < 0.05; ^#^comparison with the CXB group, *p* < 0.01; ^&^comparison with the IR plus CXB group, *p* < 0.01. **(C)** Representative images of VM formation in A549 and H460 cells transfected with non-silencing siRNA (siCon) or siRNA-ITAV (siITAV), grown on the 3D Matrigel. Scale bar, 200 μm. **(D)** Number of tubules. Values are expressed as mean ± SEM from three independent experiments. Inhibition of ITAV expression in cells (A549 and H460) by lentivirus transfection remarkably suppressed VM formation. *p < 0.05. CXB, celecoxib; IR, irradiation; ITAV, integrin alpha-V; APN, aminopeptidase N; VM, vasculogenic mimicry.

Cellular protein extraction from A549 cells was used for Western blotting analyses. Western blotting showed that IR at a dose of 5 Gy significantly increased ITAV and APN levels, while CXB treatment at a concentration of 15 μg/ml significantly decreased ITAV and APN levels (**Figure 5A**). Furthermore, IR plus CXB treatment remarkably reduced the increment of ITAV and APN levels induced by IR (**Figure 5A**). However, the protein expression of AKT1, p-AKT, and NOS3 showed no significant difference between these treatments. Thus, APN and ITAV were more likely to be the potential off-targets of CXB.

Furthermore, APN enzyme activity experiment in cells (A549 and H460) showed that, compared with the control group, IR at a dose of 5 Gy significantly increased APN enzyme activity by about 25%, while CXB reduced APN enzyme activity by about 20% (**Figure 5B**). Importantly, a combination of IR with CXB treatment significantly resulted to approximately 37% reduction in APN enzyme activity as compared with the IR alone group (*p* = 0.030 and 0.010 for A549 and H460 cells, respectively; **Figure 5B**).

Inhibition of ITAV expression in cells (A549 and H460) by lentivirus transfection remarkably suppressed VM formation (**Figures 5C, D**). This result confirmed that the suppression of ITAV expression by CXB could effectively repress the formation of VM.

### Celecoxib Inhibited Irradiation-Enhanced Vasculogenic Mimicry Formation in Xenografts

**Figure 6:**
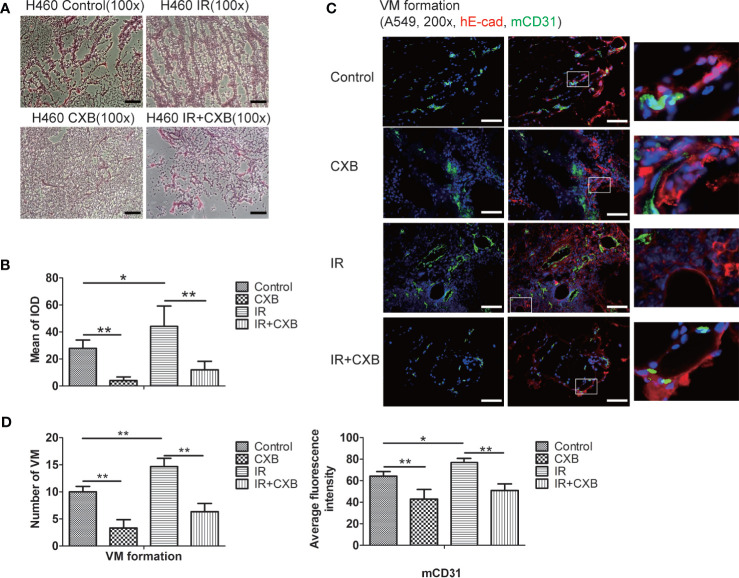
VM change in BALB/c xenografts detected by PAS (H460) and immunofluorescence staining (A549). **(A)** PAS staining (H460): vessel-like structures were composed of tumor cells and basal membrane (VM formation). VM channels were identified as vessel-like structures composed of tumor cells and were PAS-positive (red). Scale bar, 500 μm. **(B)** The mean of IOD test by PAS staining in tumor tissues. *p < 0.05, **p < 0.01. **(C)** Immunofluorescence staining of A549 cells (in A549 xenografts in BALB/c nude mice). mCD31 staining represents mouse-derived endothelial cells (green); hE-cad, a cell–cell adhesion molecule staining represents human tumor-derived basal membrane (red), and DAPI represents the cell nucleus (blue). VM channels were identified as mCD31-/hE-cad+. Scale bar, 100 μm. **(D)** The number of VM channels (left) and the expression of mCD31 (right) assessed by immunofluorescence staining in tumor tissues. *p < 0.05, **p < 0.01. VM, vasculogenic mimicry; PAS, periodic acid solution.

PAS staining showed more vessel-like structures in H460 xenografts of BALB/c mice in IR group than in the control group, while a combination of IR treatment with CXB noticeably inhibited this enhancement (**Figures 6A, B**). In A549 xenografts in BALB/c nude mice, immunofluorescence staining also showed that the formation of VM significantly increased after IR treatment, while a combination treatment of IR and CXB eliminated such increment (**Figures 6C, D**). The above results suggest that CXB can effectively block the formation of VM *in vitro* and *in vivo*, which may enhance the sensitivity of lung cancer cells and tissues to IR, supported by the remarkable different changes in tumor volume between the IR group and IR plus CXB group.

### Confirmation of Novel Targets of Celecoxib in Tumor Tissue

**Figure 7:**
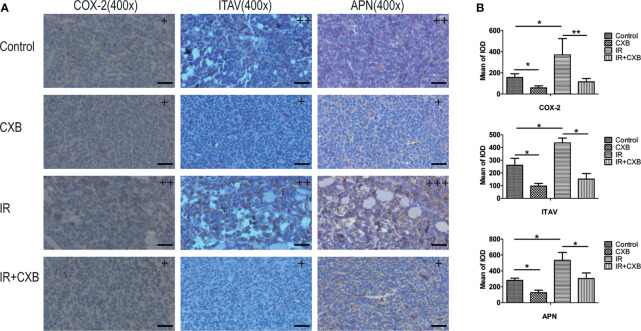
Confirmation of off-targets of CXB in LLC xenografts. **(A)** The COX-2, ITAV, and APN protein expression in tumor tissues was assayed by immunohistochemistry (the control, CXB, IR, and IR plus CXB groups). The nucleus was stained blue; and the COX-2, ITAV, and APN expression were stained brown. +, ++, and +++: The subjective scores of the tissue sections by immunohistochemical staining of COX-2, APN, and ITAV, respectively. Scale bar, 300 μm. **(B)** The mean of IOD test (the LLC xenografts in C57 mice). ^*^p < 0.05, **p < 0.01. CXB, celecoxib; LLC, Lewis lung cancer; COX-2, cyclooxygenase-2; ITAV, integrin alpha-V; APN, aminopeptidase N; IR, irradiation.

To verify the off-target effect of CXB in different animal models, the protein expression of COX-2, ITAV, and APN was confirmed in LLC xenografts. The LLC xenografts in C57 mice in the IR group showed higher COX-2, ITAV, and APN levels than those in the control group (**Figures 7A, B**), while 15 μg/ml of CXB treatment remarkably decreased these protein expression levels. Furthermore, these increments of COX-2, APN, and ITAV levels caused by IR were eliminated by CXB treatment (**Figures 7A, B**).

## Discussion

To date, IR as a treatment of NSCLC still confronted tumor relapse due to radiation resistance. In the present study, we found that IR enhanced VM *in vitro* and *in vivo*. Formation of VM channels is one of the most common causes of distant metastasis and poor prognosis in lung cancer (21, 22). Therefore, inhibiting VM channels is an important way to prevent tumor relapse and to improve clinical prognosis. Recently, several studies reported that mixed usage of chemicals and micelles exhibited destructive effects on VM channels in NSCLC, such as dequalinium modified paclitaxel plus ligustrazine micelles and paclitaxel plus honokiol micelles (34, 35). Another method to destroy VM formation in NSCLC is using liposomes (36). Since IR enhances VM *in vitro* and *in vivo*, we presume that VM is also an important factor for tumor recurrence after radiation.

One important finding in the present study was that CXB effectively eliminated VM formation in A549 and H460 cells and in murine tumor tissues; furthermore, CXB combined with IR prolonged the survival duration of mice with xenografts, consistent with its inhibitory effect on the formation of VM channels. In this study, we found that CXB as a single medicine could totally inhibit VM channel construction in NSCLC, thus contributing to its positive effect on radiosensitivity. CXB is known to play roles in suppressing inflammation, tumor growth, and angiogenesis and enhancing radiosensitivity and chemotherapy (37–39). Our findings also suggest that CXB can be used in an exciting novel disease treatment, i.e., as anti-cancer therapy or radiosensitivity promoter, apart from its traditional usage in the treatment of rheumatoid arthritis and osteoarthritis for many years.

It is well known that VM is not the only factor that affects tumor growth. The tumor vasculature is complex *in vivo*, in which re-vascularization and alternative mechanisms for re-vascularization might be taking place. So although CXB significantly inhibited the formation of VM in **Figure 2E**, CXB plus IR *in vivo* only shrunk the size of the tumor instead of banishing it as shown in **Figures 1A**, **B**. These findings suggested that CXB improved the antitumor effect of IR, benefited the prognosis, or reduced IR resistance *in vivo*. Evidence from other studies showed that CXB–erlotinib combination or CXB alone treatment can enhance radiosensitivity in A549 lung cells (40) and may be beneficial for patients with advanced NSCLC and EGFR wild type only (41). Apart from being used as a treatment of rheumatoid arthritis and osteoarthritis, CXB may hold promise of an antitumor treatment that requires further confirmation by clinical trials in the future.

As for VM identification, however, it was reported that PAS staining was not specific, and there is a lack of specific markers for VM and functional verification of tubular (10). In this study, we used not only PAS staining but also double staining in immunofluorescence. Mouse antibody mCD31 staining represents mouse-derived ECs (green); and hE-cad, a cell–cell adhesion molecule, staining represents human tumor-derived basal membrane (red) in **Figure 6**. Thus, we can identify the VM accurately. It is a novel method for VM verification in future research.

Moreover, another interesting and important finding in the present study was that CXB could inhibit not only the expression of COX-2 but also off-targets. In recent years, accumulating evidence has shown that CXB can exert anticancer effects, including promoting apoptosis, blocking cell cycles, and inhibiting angiogenesis, not relying on the COX-2 enzyme (42, 43), which means “off-target effect” of CXB. Molecular docking found that CXB could bind to the functional pocket of APN, NOS3, AKT1, and ITAV. Among these proteins, ITAV and APN had higher affinity with CXB; and the binding of CXB to ITAV and APN resulted in downregulation of these two proteins. The proteins APN and ITAV play important roles in tumor metastasis and angiogenesis (44–47). APN, also known as CD13, has been reported to be related to poor prognosis when expressed in tumor tissues (48, 49). Recently, ITAV level in cancer tissue has been found positively correlated with tumor invasion and metastasis, such as in prostate cancer and osteosarcoma (50, 51). Thus, these proteins involved in tumor metastasis may have a strong relationship with VM, or participate in VM formation. Unfortunately, seldom studies reported the roles of these proteins in the formation of VM. One cellular study revealed that EGFR–integrin αvβ3 complex impairment repressed VM in triple-negative breast cancer (52).

In the current study, vessel-like structures were formed in A549 and H460 cells with higher APN expression, suggesting that APN may participate in VM formation. Consistent with the effect of IR and CXB on VM formation, IR significantly increased APN levels in cells and mice, while CXB outstandingly decreased APN levels. Furthermore, CXB inhibited the increment of APN enzyme activity in cells. These findings indicated that CXB can be an inhibitor of APN and may be used for lung cancer treatment in clinical practice like other aminopeptidase inhibitors such as bestatin (53). Recently, one clinical study including 270 patients with NSCLC revealed that vascular CD13 protein expression was correlated with poor overall survival in stage III and pN2+ NSCLC patients (54). To our best knowledge, no study has reported that aminopeptidase and its inhibitors play a role in VM formation. The present study provides moderate evidence that APN is involved in the formation of VM.

ITAV was another potential off-target of CXB in VM formation, as demonstrated by the current study. ITAV is one member of a family of cell adhesion proteins. Similar to the results of APN, IR significantly increased ITAV levels in A549 and H460 cells and in mice, while CXB remarkably decreased ITAV protein levels. Additionally, the increments of ITAV levels caused by IR in the tumor tissues of C57 mice were eliminated by CXB treatment. Furthermore, inhibition of ITAV expression by CXB and transfection of lentivirus into tumor cells induced a notable reduction of VM formation. These results identified a positive important role of ITAV in VM formation, which is a novel finding in lung cancer. Inhibition of ITAV by intetumumab, an anti-ITAV monoclonal antibody, has shown anti-migratory and anti-proliferative effects *in vitro* and therapeutic and preventive effects *in vivo* (55–58).

To our best knowledge, this is the first study to report that CXB could inhibit VM in radiosensitizing lung cancer. It is also the first study demonstrating that CXB has off-targets in the process of destroying VM. There were some limitations in this study. First, PAS staining is not specific for VM verification. Thus, we did mCD31/hE-cad double staining immunofluorescence to provide direct evidence of VM formation as a quantified method. Second, the mechanism of off-targets APN and ITAV should be clarified in the future. Whether CXB acts as a specific inhibitor of APN or ITAV to inhibit VM formation needs to be explored.

## Conclusions

Collectively, CXB enhances radiosensitivity in NSCLC *in vitro* and *in vivo* through inhibition of VM formation involving downregulating COX-2 and novel targets APN and ITAV. This is an off-target effect of CXB and may provide new insights into the mechanisms underlying VM formation. Our findings hold promise of an effective strategy for eliminating IR resistance in NSCLC.

## Data Availability Statement

The original contributions presented in the study are included in the article. Further inquiries can be directed to the corresponding authors.

## Ethics Statement

The animal study was reviewed and approved by the Laboratory Animal Welfare and Ethics Committee of Army Medical University.

## Author Contributions

KN, XWC, and LPZ carried out the experiments. KN and XWC analyzed the data. RXL and JGS conceived and designed the experiments. KN and YQ drafted the manuscript. All authors contributed to the article and approved the submitted version.

## Funding

This study was funded by the National Natural Science Foundation of China (Nos. 81773245, 81972858), Technology Innovation and Application Development Project of Chongqing (No. cstccxljrc201910), the Cultivation Program for Clinical Research Talents of Army Medical University in 2018 [2018XLC1010], and the Science and Technology Innovation Special Project of Chongqing Social Undertakings and Livelihood Security (No. cstc2017shmsA130108).

## Conflict of Interest

The authors declare that the research was conducted in the absence of any commercial or financial relationships that could be construed as a potential conflict of interest.

## Publisher’s Note

All claims expressed in this article are solely those of the authors and do not necessarily represent those of their affiliated organizations, or those of the publisher, the editors and the reviewers. Any product that may be evaluated in this article, or claim that may be made by its manufacturer, is not guaranteed or endorsed by the publisher.
